# Goal orientations of health profession students throughout the undergraduate program: a multilevel study

**DOI:** 10.1186/s12909-016-0621-5

**Published:** 2016-03-31

**Authors:** Ada Kool, Tim Mainhard, Mieke Brekelmans, Peter van Beukelen, Debbie Jaarsma

**Affiliations:** Faculty of Social and Behavioural Sciences, Utrecht University, P.O. Box 80140, 3508 TC Utrecht, Netherlands; Faculty of Veterinary Medicine, Utrecht University, Utrecht, Netherlands; Center for Research and Innovation in Medical Education, University Medical Center Groningen, Groningen, Netherlands

## Abstract

**Background:**

The achievement goal theory defines two major foci of students’ learning goals (1) primarily interested in truly mastering a task (mastery orientation), and (2) striving to show ones competences to others (performance orientation). The present study is undertaken to better understand if and how health profession students’ goal orientations change during the undergraduate program and to what degree gender, academic achievement, and self-efficacy are associated with mastery and performance orientation *between* students and *within* students over time.

**Method:**

By means of an online questionnaire, students of medical, pharmaceutical, and veterinary sciences (*N* = 2402) were asked to rate themselves on mastery orientation, performance orientation, and self-efficacy at the beginning of five consecutive semesters. Data on grades and gender were drawn from university’s files. Multilevel analyses were used for data analysis.

**Results:**

Students’ goal orientations showed relative stability over time, but substantial fluctuations *within* individual students were found. These fluctuations were associated with fluctuations in self-efficacy. Students’ gender, high school grades, study grades, and self-efficacy were all associated with differences in mastery or performance orientation *between* students. Self-efficacy was the strongest predictor for mastery orientation and grades for performance orientation.

**Conclusions:**

The relatively strong association between the goal orientations and students’ self-efficacy found in this study emphasizes the potential of enhancing self-efficacy in health profession students. Also, for educators and researchers, fluctuations of both goal orientations *within* individual students are important to consider.

## Background

According to the achievement goal theory, two major foci of students’ learning goals are 1) being primarily interested in learning and in truly mastering a task (mastery orientation), and 2) striving to outperform peers and showing ones competences to others (performance orientation) [1, 2]. Students can endorse both goals at the same time [3], and may revise their goals as they progress in their study [4, 5]. Educational researchers refer to achievement goals as the reason why students engage in a task. Moreover, the achievement goal theory is one of the most important frameworks to analyse student motivation and to study its effects on learning and performance. The type of goal students pursue influences their learning outcomes and behaviour [3]. Mastery goals have been linked to continued interest in the subject [6], persistence in the face of obstacles [7], self-regulated learning [8], help-seeking behaviour [9], and the use of deep processing strategies [10]. Orientation towards performance is associated with the use of more shallow processing strategies [10], but also higher academic performance [11]. In the health professions domain both goal orientations have a significant function, since professionals are expected to focus on performance for the sake of their patients, while continuously need to learn new skills [12]. Knowledge on how and why students’ goal orientations change during the study program and why students differ in their goal orientations provides educators with insights in how to strengthen a specific type of motivation in their students. For students, it is important to gain insight in how to regulate their goal orientations in the light of lifelong learning and for the good of their future patients. The present study was undertaken to investigate if and how health profession students’ goal orientations change during the first three study years (undergraduate bachelor program) and what constructs drive this process.

Studies outside the health professions domain suggest that students’ goal orientations remain stable or decline during the first year at university [13, 14]. Fryer and Elliot [5] found that mastery orientation declined, while performance orientation remained stable within one academic year in a sample of psychology students. Within the health professions domain, hardly any studies on this topic have been conducted. An exception is a study conducted by Artino and colleagues [15], who showed that first- and third –year medical students were more mastery oriented compared to second-year students, while students did not differ in their performance orientation. This suggests that mastery orientation of medical students fluctuates somewhat over time, however this study’s design was cross-sectional, making it hard to draw firm conclusions on developmental issues. Further, Senko and Harackiewicz [16] noted that goal orientations can change either by *intensification*, representing an increase or decrease of a single goal orientation or multiple goals in the same direction, or by *switching*, which refers to a concurrent decrease in one goal orientation and an increase in the other. Knowledge on possible fluctuations in goal orientations has important theoretical implications, since conclusions regarding the effect of goal orientations on students study behaviour and outcome measures (e.g., academic achievement) may partly depend on when those goal orientations are measured. Our study adds to the present literature by examining stability and change in goal orientations both independently and in relation to each other by using a sample of health profession students over time.

Previous studies examined factors that may explain differences in goal orientations *between* students and *within* students over time [5, 16]. This distinction is important, since both approaches provide different insights. Differences between students may provide insight in relations between goal orientations and students’ personal characteristics, while differences within students refer to the question whether the strength of goal orientations can actually change over time and how this change can be achieved. In literature, three prevalent constructs that have been related to differences in goal orientation are gender, academic achievement, and self-efficacy (i.e., the extent or strength of a students’ belief in their own ability to complete certain tasks) [16–18]. Male students are generally more performance orientated, while female students are more mastery oriented [19]. Similarly, students with a higher study Grade Point Average (GPA) show higher levels of mastery and performance orientation [11]. Also, students with a high self-efficacy, thus those who expect to perform well, report higher levels of mastery and performance orientation [20–22] as compared to students with lower self-efficacy. The relation between both academic achievement and self-efficacy with goal orientations however seems to be complex. Self-efficacy has been found to be a direct predictor of goal orientations, but also a moderator on the relation between academic achievement and goal orientations [18]. Moreover, academic achievement has been studied as an outcome of goal orientations [11, 18], but also as a predictor of goal orientations [18]. Fluctuations in goal orientations *within* students over time may be related to changes in academic achievement and self-efficacy through time within a single student. For example, a students’ level of self-efficacy may change during the academic year due to exam failure [16], subsequently causing fluctuations in levels of mastery and performance orientation or vice versa. Thus, self-efficacy and academic achievement are important associates of goal orientations *between* and *within students* and may be a framework for the development of interventions. In this study, we only investigate direct relations between self-efficacy and previous academic achievement with goal orientations.

Up to now, little research has focused on correlates of *within-student* fluctuations of goal orientations [5]. In the current study, multilevel analyses were used to examine to what degree stable as well as time varying variables are associated with differences in mastery and performance orientation among students (between-student differences) as well as with fluctuations within individual health profession students over the undergraduate bachelor program (within-student differences). The following research questions are addressed:

i) How do mastery and performance orientations change in health profession students (medical sciences, pharmaceutical sciences, and veterinary sciences) over the course of the undergraduate bachelor program?
ii) To what degree do gender and (changes in) academic achievement, and self-efficacy explain differences in mastery and performance orientation *between* students and *within* students over time?

## Methods

### Participants and procedure

This study involves students from three undergraduate professional programs: medical sciences, pharmaceutical sciences, and veterinary sciences at Utrecht University, The Netherlands. The professional programs offer a three-year (undergraduate) bachelor program, followed by a two-year (graduate) master program for pharmaceutical sciences and a three-year master program for medical- and veterinary sciences. Each undergraduate academic year is divided into two semesters consisting of multiple courses. Although the bachelor curricula of all three programs include basic science knowledge, but also clinical science knowledge and practical skills, organization of the curriculum differs between the programs.

Students in this sample were drawn from a dataset containing data on student self-perceptions over time including 12094 students from sixteen different undergraduate programs at Utrecht University. The first author obtained the primary dataset. All undergraduate students of the involved undergraduate programs were invited to participate in an online questionnaire at the beginning of five consecutive semesters starting in March 2010 and ending in March 2012. Thus students from different cohorts were followed during (a part of) their undergraduate program between 2010 and 2012. This implies that students who were already in their third undergraduate year at the time of the first data collection could participate only once, while students who were in their first year at that time received an invitation for all five data collections.

For the present study, the sample consisted of 2402 individual students (42 % medical sciences, 33 % veterinary sciences, and 25 % pharmaceutical sciences; 75 % female respondents) who together completed 4910 questionnaires (students participated one (*N* = 2258), two (*N* = 1129), three (*N* = 835), four (*N* = 442) or five (*N* = 246) times (response rate data collection 1 = 53 %, 2 = 56 %, 3 = 43 %, 4 = 37 %, 5 = 37 %).

### Ethics, consent and permissions

At the start of the original study, in 2010, no ethical committee was established at the institution where the study was conducted. However, we ensured alignment with the rules of the Helsinki declaration by obtaining written, informed consent, guaranteeing confidentiality, and presenting all data anonymously. Participation was on voluntary basis and participants could withdraw from the study at any time without explanation. In addition, retrospective approval for the study was provided by the Ethics Committee of the Faculty of Social and Behavioural Sciences of Utrecht University, after the committee was established.

### Instruments

The online questionnaire designed included existing, validated subscales to measure mastery orientation, performance orientation, and self-efficacy. Factor analyses were conducted on the scales of professional excellence and the scales included in the propensity model to test for scale uni-dimensionality, using maximum likelihood with oblique rotation as an extraction method [23]. Scale reliability was established. All items used in the present study can be found in the Appendix.

#### Goal orientations

Mastery (α = .73) and performance goal orientation (α = .88) were measured using scales of the Achievement Goal Questionnaire (AGQ; [2]). This questionnaire is a widely applied instrument to assess mastery and performance goal orientation [24]. Both scales consisted of three items on a 7-point Likert scale, ranging from 1 (not at all true of me) to 7 (very true of me). A mastery score and a performance score were calculated per student and per semester by averaging the three items.

#### Self-efficacy

Self-efficacy (α = .65) was measured with three items on a 7-point Likert scale using the PISA index of general academic self-efficacy [25]. As an indicator of a students’ general self-efficacy we calculated the average across the entire research period for each student and as an indicator of fluctuations in self-efficacy *within* individual students we calculated the semester specific deviation from a student’s average self-efficacy.

#### Academic achievement

Grade Point Averages (GPA) were used as a measure of academic performance. With active informed consent of all participants, students’ responses were linked with high-school grades, and university test grades all drawn from the university’s files. For each student, average study GPA was calculated by averaging all grades obtained during the professional program up until the moment of data collection, corrected for the amount of credits per course. Also for each student, semester specific GPA was calculated by averaging all grades obtained during the semester previous to the questionnaire data collection. This semester specific GPA was converted into deviation-scores (deviation of the semester GPA from the average GPA) in order to measure fluctuations *within* individual students through time. High-school GPA was included as a measure of academic performance at university entrance.

In the Netherlands, GPA is a weighted average calculated with pass-marks only. Grades range from 1 (lowest) to 10 (highest), but in order to pass an exam a score of at least 5.5 is required. In our sample study GPA ranged between 5.99 and 9.44, M = 7.1, SD = 0.65. High-school GPA ranged between 5.78 and 9.60, M = 7.29, SD = 0.69.

### Data analysis

Given the rather uncommon dataset used in the current study, a design that combines cross-sectional with longitudinal data, we employed complex multilevel modelling analyses that have not been used much in medical education (but see for example [26]). An advantage of multilevel longitudinal modelling is that it accounts for differences between students in number of times they participated. This way, all participants contribute to the estimation of the tested models [27].

Multilevel analysis further allows decomposing the variance in goal orientations into variance *between* students and variance *within* students [27]. Variance *between* students indicates how different students differ in their goal orientations, while variance *within* students refers to how individual students fluctuate in their goal orientations over time. A next step is to explain potential differences in goal orientations between students or within students over time by the inclusion of explanatory variables (e.g., gender or a students’ self-efficacy). In this study we used gender, high-school GPA, overall study GPA, and average self-efficacy of a student to explain potential differences between students (see Table 1 for descriptives). In order to explain differences in goal orientations within individual students over time we used students’ fluctuations in self-efficacy and in GPA over time (see [28]).

**Table 1:** Descriptives of study variables. *N* = 2402 individual students, 4910 measurement occasions

	M	SD	Minimum	Maximum
Mastery orientation (1–7)	5.28	0.87	1.33	7.00
Performance orientation (1–7)	4.24	1.39	1.00	7.00
Self-efficacy (1–7)	4.87	0.86	1.33	7.00
Study GPA (1–10)	7.05	0.54	6.00	9.50
High-school GPA (1–10)	7.35	0.69	5.78	9.60

We modelled mastery and performance orientation together in one multivariate model as two aspects of goal orientation. This way, it was possible to examine whether both goal orientations are similarly affected by our explanatory variables while accounting for the correlation of the two goal orientations. This strategy thus makes it possible to investigate the relatedness of the two goal orientations through time and if goal switching occurs. We followed the analysis strategy for multilevel analysis as described by Hox [27] fitting three subsequent multilevel models. (1) The first model comprised an ‘empty’ or variance decomposition model with only mastery and performance orientation as dependent variables and no explanatory variables added to the model. (2) In order to answer the first research question on how both goal orientations change during the undergraduate program, time was added to the model as an explanatory variable as a second step. (3) Finally, the third model was fitted which also included the other explanatory variables (i.e., gender, achievement, self-efficacy) in order to answer the second research question on whether the explanatory variables explain differences in mastery and performance orientation *between* students and *within* students over time.

Since our sample comprised three different professional programs, the professional program was entered as a control variable into the model. Whether the model improved by adding the explanatory variables was tested with a Chi-square tests on the deviance of the multilevel models (i.e., a measure of discrepancy between the data and the model; [27]). Analyses were conducted in MlWiN (Version 2.27).

## Results

When entering a health profession study, the average student is somewhat more oriented on mastery (M = 5.43) than performance (M = 4.45), with more dispersed scores for performance (SD = 1.34) than for mastery (SD = 0.83) orientation. To investigate how mastery and performance orientations change over the course of the undergraduate bachelor program and to what degree the gender, academic achievement, and self-efficacy explain differences in the goal orientations *between* and *within* students, three subsequent multilevel models were fitted. The first model, an ‘empty’ model, was required in order to decompose the variance in goal orientations into variance *between* students and variance *within* students over time. The model showed that for mastery and performance orientation respectively 56 % and 69 % of the variance was due to differences *between* students and 44 % and 31 % was due to differences *within* students over time. This indicates that goal orientations of individual students fluctuate considerable from semester to semester. However, looking at the *average* levels of mastery and performance orientation per semester, both goal orientations remained relatively stable over time (see Table 2).

**Table 2:** Levels of mastery and performance orientation over time (raw data). *N* = 2402 individual students, 4910 measurement occasions

	Mastery (1–7)	Performance (1–7)
	M (SD)	M (SD)
Semester 1 (*N* = 674)	5.43 (0.03)	4.45 (0.05)
Semester 2 (*N* = 874)	5.36 (0.03)	4.31 (0.05)
Semester 3 (*N* = 648)	5.21 (0.03)	4.13 (0.06)
Semester 4 (*N* = 915)	5.27 (0.03)	4.23 (0.04)
Semester 5 (*N* = 643)	5.12 (0.04)	4.08 (0.06)
Semester 6 (*N* = 991)	5.28 (0.03)	4.24 (0.04)

To examine changes in mastery and performance orientation over time (research question 1), a second model was fitted with time added to the model as an explanatory variable. This multilevel model including time confirmed that on average both types of goal orientation were relatively stable. The model showed that both goal orientations decreased slightly over time, but the decrease decelerated at the end of the third year (∆χ^2^(7) = 294; mastery: linear *B* = -0.17, *p* < 0.01, quadratic *B* = 0.02, *p* < 0.01; performance: linear *B* = -0.17, *p* < 0.01, quadratic *B* = 0.02, *p* < 0.01). Results of the model also showed that students differed somewhat in how they change over time (mastery: slope variance = 0.01, *p* < .001; performance: slope variance = 0.03, *p* < .01). According to the model, a student entering the professional program with a mastery orientation with an average score of 5.55, may at the end of the third year have a mastery orientation score between 3.81 and 6.69. This difference resembles more than three times the SD in mastery orientation. Similarly, for a student entering a professional program with an average performance orientation score of 4.51, after three years of study, performance orientation may have increased to 6.49 or may have declined to 1.93. Again, this range equals more than three SD in performance orientation. Changes in mastery and performance orientation over time were significantly correlated with each other (*r* = 0.45).

### Explaining differences in goal orientations

In order to answer the second research question on to what degree gender, academic achievement, and self-efficacy explain differences in the goal orientations *between* and *within* students, a third model was fitted. In this model, the three explanatory variables as well as time were added. Students’ gender, high school GPA, study GPA, and general self-efficacy were all associated with differences *between* students in mastery and performance orientation (for an overview see Table 3). Students’ fluctuations in self-efficacy were also related to differences in goal orientations *within* students over time. Grades obtained during the previous semester were not related to individual students’ goal orientations. Adding these explanatory variables largely improved the model. This indicates that the explanatory variables indeed explain a considerable amount of variance in goal orientations.

**Table 3:** Predictors of mastery and performance orientation of undergraduate health profession students: fixed effects of the multivariate multilevel growth model

	Mastery	Performance
	B/SE (β)	B/SE (β)
Intercept	5.39**	4.47**
Time	-0.21/0.04 (-0.42)**	-0.19/0.05 (-0.24)**
Time squared	0.02/< 0.01 (0.21)**	0.02/< 0.01 (0.13)**
*Between students*		
Gender	0.20/0.04 (0.10)**	ns
High school GPA	-0.13/0.04 (-0.10)**	0.15/0.06 (0.07)**
Pharmaceutical Sciences^a^	ns	0.29/0.08 (0.09)**
Veterinary Sciences^a^	0.19/0.04 (0.10)**	ns
Self-Efficacy	0.27/0.02 (0.27)**	0.32/0.03 (0.20)**
Study GPA	0.25/0.06 (0.16)**	0.58/0.09 (0.23)**
Time* Study GPA	0.05/0.02 (0.70)**	0.07/0.02 (0.62)**
*Within students*		
Self-Efficacy_dev	0.23/0.02 (0.11)**	0.21/0.03 (0.06)**
Study GPA_dev	ns	ns
Δχ^2^ (deviance)	-6472.01	

*Note.* B refers to unstandardized effects, SE refers to the standard errors of these effects, and β refers to the standardized effect. Between student variance for mastery and performance was 0.576** and 1.352**. Within student variance was 0.259** and 0.458**

* *p* < .05; ** *p* < .01 (all tested one-sided)

a reference group is Medical Sciences

#### Mastery orientation

On average, female students were more mastery oriented than male students (*B* = 0.20, *p* < 0.01, β = 0.10) and higher performing students reported to be more mastery orientated compared to their peers (*B* = 0.25, *p* < 0.01, β = 0.15). A high study GPA also tended to buffer against a decline in mastery orientation over time (*B* = 0.05, *p* < 0.01, β = 0.70). Thus, students with a high GPA in general showed a smaller decline in mastery orientation over time. Also, veterinary students showed more mastery orientation than students from the other professional programs (*B* = 0.19, *p* < 0.01, β = 0. 10). General self-efficacy of a student was the strongest associate of mastery orientation (*B* = 0.27, *p* < 0.01, β = 0.27). Students with a general self-efficacy of +1 SD reported on average a 0.51 point higher mastery orientation (about ½ SD) as compared to students with a self-efficacy of -1 SD. High-school GPA was negatively associated with mastery orientation (*B* = -0.13, *p* < 0.01, β = -0.10). The level of mastery orientation at the start of the study was related to the decline in mastery over time; students entering the professional program with higher levels of mastery orientation tended to show a greater decline over time (intercept-slope r = -0.57; Table 4).

**Table 4:** Variances (diagonal) of mastery and performance orientation of undergraduate health profession students and their correlations (off-diagonal): student level random effects of the multivariate multilevel growth model

	1. Initial mastery	2. Slope mastery	3. Slope performance	4. Slope performance
1. Initial mastery	0.576**			
2. Slope mastery	-.57**	0.018**		
3. Initial performance	.38**	ns	1.352**	
4. Slope performance	-.60**	ns	-.47**	0.027**

* *p* < .05; ** *p* < .01

Fluctuations in mastery orientation *within* students over time were positively related to students’ fluctuations in self-efficacy (*B* = 0.23, *p* < 0.01, β = 0.11). Grades obtained during the previous semester were not related to the level of subsequent mastery orientation.

#### Performance orientation

A higher self-efficacy was associated with higher scores on performance orientation (*B* = 0.32, *p* < 0.01, β = 0.20). Also, study GPA was positively associated with performance orientation (*B* = 0.58, *p* < 0.01, β = 0.23). According to our model, students with a study GPA of +1 SD were 0.61 point (about ½ SD in performance orientation) more performance oriented as compared to students with a study GPA of -1 SD. Students with a relatively higher study GPA tended to show a smaller decline in performance orientation over time (*B* = 0.07, *p* < 0.01, β = 0.62). A higher high-school GPA tended to be positively associated with performance orientation too (*B* = 0.15, *p* < 0.01, β = 0.07). Pharmaceutical science students were in general somewhat more performance orientated compared to their peers from medical and veterinary sciences (*B* = 0.29, *p* < 0.01, β = 0.09).

Students entering the professional program with high levels of performance orientation were more likely to decline in performance orientation over time (*r* = -0.47; Table 4). Interestingly, higher levels of *mastery* orientation at the start of the study in general also showed a greater decline in performance orientation (*r* = -0.60).

Fluctuations in performance orientation *within* students over time were slightly positively associated with fluctuations in self-efficacy (*B* = 0.21, p < 0.01, β = 0.06). When students’ self-efficacy was higher than usual, the level of performance orientation tended to increase as well. Grades obtained during the previous semester were not related to subsequent performance orientation.

## Discussion

In this study we investigated the stability and change of health profession students’ mastery and performance orientation over the undergraduate program and whether study GPA, high school GPA, self-efficacy and gender are associated with this process. The use of multilevel analyses and the accelerated longitudinal design of this study provided insight on correlates of differences in goal orientations *between* students and *within* individual students over time. It is important to gain knowledge on the stability and changes of goal orientations, since goal orientations are associated with various aspects of learning and behaviour (e.g., [10, 11]). Also, identifying constructs that are associated with fluctuations in goal orientations provide educators insight in how to strengthen their students’ goal orientations and for example how to slow down possible declines of goal orientations over time.

According to our results, students differ largely in their goal orientations. The overall conclusion regarding the first research question on stability and change of the goal orientations over time is that the average levels of mastery and performance orientations do not increase or decline across the undergraduate professional programs, however individual students fluctuate quite substantially in their reported goal orientations from semester to semester.

In line with this, since the two goal orientations correlated positively no support for goal switching was found (see also [5, 11]), which indicates that students did not increase the level of one goal orientation at the expense of the other goal orientation. The average stability, with only a small decline in both goal orientations during the undergraduate program, was in line with former studies [13, 14, 29]. This implies that students entering the professional program with relatively low levels of the goal orientations are not likely to experience an overall gain in these orientations. In addition to the relatively stable average trend over time across students we found rather substantial fluctuations *within* students, which accounted for more than one-third of the variance in goal orientations. This is an important finding with implications for future research, since such fluctuations are not captured when using cross-sectional data, only one or two measurement points, or when only average trends are calculated. Moreover, the substantial fluctuations in levels of the goal orientations *within* individual students are also relevant to consider for health profession educators. Individual students’ mastery and performance orientation seems to decline and increase during the academic year. This indicates that goal orientations change according to the learning environment and experience.

The second research question was aimed at providing insight in constructs associated with these fluctuations *within* individual students as well as differences in goal orientations *between* students. Self-efficacy indeed occurred to be an important correlate of both differences in levels of goal orientations *between* students and *within* student fluctuations, especially for mastery orientation. Students with a high average level of self-efficacy showed higher levels of mastery and performance orientation. Also for individual students, when self-efficacy in a student peaked, levels of both goal orientations were also high. This highlights the importance of enhancing self-efficacy in health profession students. Our results confirm previous research from general education on this topic [21, 22] for the health professions domain.

Additionally, high-school GPA was associated with differences between students. Students with a relatively higher high-school GPA were generally less mastery oriented and more performance oriented as compared to lower-achieving peers. These findings are particularly relevant to the health profession domain, since there is a lot of emphasize on high-school grades in many selection procedures of health profession studies [30]. Selecting on the basis of high-school grades may thus possibly bias the student population towards a performance orientation. The question arises whether this is what health educators aim for. Although a performance orientation is certainly useful in in health care professions [12], the importance of a life-long learning approach is essential in becoming an expert health professional too [31]. Moreover, the strength of mastery orientations at the beginning of the professional program was also substantially associated with how performance orientation developed through time. A stronger mastery orientation at the start of the professional program buffered to a certain degree a decline in performance orientation.

Interestingly, our study did not confirm that *within students,* changes in academic achievement are associated with subsequent changes in goal orientations see for example [11]). Possibly students’ self-efficacy acts not only as a direct predictor for goal orientations, but also as a mediator between academic achievement and goal orientations. For example, Diseth [18] found in a sample of psychology students that academic achievement influenced self-efficacy, which in turn predicted mastery and performance orientation (see also [32]). In order to identify what students need to enhance their learning, more research on the relations between academic achievement, goal orientations, and self-efficacy in the medical education context is required.

Finally, it is important to consider that students entering the professional program with high mastery or performance orientation experienced a greater decline over time. Possibly, highly motivated students enter the professional program with somewhat exaggerated expectations. Studies in the health profession are intense and require hard work, adequately informing future students about the content and workload of the professional program may prevent a decline in motivation as a result of unfulfilled expectations.

### Study limitations

Although our sample included three largely independent health profession programs, the study had a single-institution design. Also, students participated in the study on voluntary basis and it is not clear whether the results can be generalized to students who decided not to participate. However, study GPA range and mean of our sample (range = 6.0-9.1, *M* = 7.0) was very similar to that of all invited students (range = 6.0-9.4, *M* = 6.9).

Further, we used self-reports, which may have resulted in a certain bias, for example, students may have responded in a socially desirable [33] or self-serving way. We tried to diminish this by guaranteeing the participants that questionnaires would only be used for research purposes. Also, the reported ranges in mastery and performance goals do not seem to indicate a bias towards reporting exaggerated levels of motivation.

This study was designed as a first step toward more understanding on changes and stability of goal orientations over time using a large, heterogeneous sample. Future studies examining goal orientation over time in relation with other contextual and performance assessment data and in smaller, more homogeneous samples would be a valuable addition to the present study.

Students who decided to complete only one measurement occasion may have different levels of goal orientations than their peers who participate in all subsequent measurements. We checked whether there was a correlation between the ratio of participation (number of times a student participated divided by the maximum number a student possibly could have participated) and the goal orientations. There was no correlation between the ratio of participation and performance orientation. There was a very small, but significant (*r* = 0.06, *p* < 0.01) correlation between the ratio of participation and mastery orientation. It should therefore be noted that our sample included students who were slightly more mastery oriented than the overall sample.

Another issue we would like to raise is that mastery and performance orientation are also used in a 2×2 framework [2], in which both goal orientations are sub-divided into approach and avoidance components. In this study we focused on the more positive approach components, following other recent studies in the educational field [11] and related constructs in medical education literature [19]. However, we are not certain how including the avoidance components would have changed the results. Further, we used validated scales for mastery orientation, performance orientation, and self-efficacy. The scales also proved to be reliable in our sample. However, as there are more, and also more elaborate, scales available, other scales may lead to deviant results. Nonetheless, in general our findings were in line with earlier research in professions other than health-care (e.g., [14, 29]).

### Implications

Results of this study yield several practical implications for health profession education. Fluctuations of goal orientations within students over time indicate that students’ goal orientations actually change during the academic years. For individual students, an important first step in the regulation of their goal orientations is awareness that their goals are actually fluctuating. Educators can increase this awareness in students, and further guide them in regulating their goal orientations. Moreover, the adaptive nature of the goal orientations also provides opportunities for health profession educators to facilitate the adoption of a mastery or performance orientation by students through the way they provide instructions to students [15]. Emphasising the importance of performance outcomes, stimulating competition between students, and testing students frequently would lead to a performance orientation in classrooms. On the other hand, a mastery orientation can be supported by tasks and feedback promoting creativity and risk taking, emphasising the importance of truly understanding the material, and providing formative feedback focusing on progress made by students [34].

For health profession researchers, results show that studies addressing mastery and performance orientation should be aware that outcomes partly depend on *when* the goal orientations are measured. The use of multilevel analyses with at least three moments of measurement and the inclusion of random slopes when examining goal orientations of health profession students over time is therefore strongly recommended.

The study showed that self-efficacy was an important correlate of differences in goal orientations *between* and *within* students. This indicates that we should attempt to enhance and stabilize self-efficacy in health profession students. When students learn to regulate their self-efficacy, this would benefit their levels of mastery and performance orientation when working as individual health professionals. High levels of the goal orientations supports lifelong learning in the health profession domain and leads to improved performance [12]. According to Bandura [17] students base their self-efficacy on four major sources of information: previous performances, vicarious experience, verbal persuasion, and psychological state. By providing positive and constructive feedback, teachers can therefore directly influence a student’s self-efficacy. This type of feedback focuses on the process of learning. Teachers can provide information on the progress that has been made and show the gap between current knowledge and the desired end goal [35].

Finally, it seems that especially students who show high initial levels of the goal orientations at the start of the study are not able to maintain their levels of the goal orientations throughout the study. This underpins the importance of identifying those students who increase in their emphasis on mastery and performance orientation in order to delineate those factors that motivate students.

## Conclusion

Although mastery and performance orientation are relatively stable across the undergraduate professional programs, individual students differ largely in their goal orientations. Students’ gender, high school grades, study grades, and self-efficacy are all associated with differences in mastery or performance orientation *between* students. Also, *within* students quite substantial fluctuations in their reported goal orientations occur from semester to semester. These fluctuations are associated with a students’ level of self-efficacy.

### Availability of data and materials

Questionnaire items used are available in the appendix. The complete dataset can be requested from the first author.

## **Appendix**

Overview of the questions used to measure performance orientation, mastery orientation, and self-efficacy

Performance orientation

1. It is important for me to do better than other students
2. It is important for me to do well compared to others in this class
3. My goal in this class is to get a better grade than most of the other students

Mastery orientation

1. I want to learn as much as possible in my study
2. It is important for me to understand the content of my courses as thoroughly as possible
3. I desire to completely master the material presented in my study

Self-efficacy

1. I am certain I can understand the most difficult material presented in texts
2. I am confident I can do an excellent job on assignments and tests
3. I am certain I can master the skills being taught

## Abbreviations

GPA: Grade Point Average
